# ﻿Chromosome complements of *Channalucius* and *C.striata* from Phu Quoc Island and karyotypic evolution in snakehead fishes (Actinopterygii, Channidae)

**DOI:** 10.3897/compcytogen.v17.i1.94943

**Published:** 2023-01-03

**Authors:** Denis V. Prazdnikov

**Affiliations:** 1 Severtsov Institute of Ecology and Evolution, Russian Academy of Sciences, Leninsky pr. 33, Moscow, 119071, Russia Severtsov Institute of Ecology and Evolution, Russian Academy of Sciences Moscow Russia

**Keywords:** *Channa* species, chromosomal rearrangements, karyotype differentiation, snakeheads

## Abstract

Snakehead fishes of the family Channidae are obligatory air-breathers freshwater predators, the vast majority of which belong to the genus *Channa* Scopoli, 1777. *Channa* species are characterized by high karyotypic diversity due to various types of chromosomal rearrangements. It is assumed that, in addition to the lifestyle, fragmentation and isolation of snakehead populations contribute to an increase in karyotypic diversity. However, the chromosome complements of many isolated populations of widespread *Channa* species remain unknown, and the direction of karyotype transformations is poorly understood. This paper describes the previously unstudied karyotypes of *Channalucius* (Cuvier, 1831) and *C.striata* (Bloch, 1793) from Phu Quoc Island and analyzes the trends of karyotypic evolution in the genus *Channa*. In *C.lucius*, the karyotypes are differed in the number of chromosome arms (2n = 48, NF = 50 and 51), while in *C.striata*, the karyotypes are differed in the diploid chromosome number (2n = 44 and 43, NF = 48). A comparative cytogenetic analysis showed that the main trend of karyotypic evolution of *Channa* species is associated with a decrease in the number of chromosomes and an increase in the number of chromosome arms, mainly due to fusions and pericentric inversions. The data obtained support the assumption that fragmentation and isolation of populations, especially of continental islands, contribute to the karyotypic diversification of snakeheads and are of interest for further cytogenetic studies of Channidae.

## ﻿Introduction

The family Channidae includes two genera of freshwater snakehead fishes (*Parachanna* Teugels et Daget, 1984 and *Channa* Scopoli, 1777) with a disjunct range (Courtenay and Williams 2004; Rüber et al. 2020). The genus *Parachanna* is restricted to tropical Africa and contains three species. The genus *Channa* is more numerous in terms of the number of species (more than 40 species described to date) distributed mainly in Southern Asia (Fricke et al. 2022; Froese and Pauly 2022). The estimated number of species in this genus varies as the group is subject to frequent taxonomic revisions and the currently accepted nominal species may constitute species complexes (Adamson et al. 2010; Cioffi et al. 2015; Conte-Grand et al. 2017).

Appearing in the Eocene, snakehead fishes have undergone a long evolution with multiple range expansions and repeated contacts with lineages that had diverged in isolation (Adamson et al. 2010; Rüber et al. 2020), reflected in their karyotypic diversity. Among the cytogenetically studied *Channa* species, the number of chromosomes varies from 2n = 32 to 2n = 112, while the number of chromosome arms from NF = 46 to NF = 116 (Kumar et al. 2019). Given such high karyotypic diversity, it is obvious that the evolutionary dynamism in the genus *Channa* is a result of various types of chromosomal rearrangements, the main of which are pericentric inversions, fusions, and polyploidization (Dhar and Chatterjee 1984; Rishi and Haobam 1990; Tanomtong et al. 2014; Cioffi et al. 2015). At the same time, the trends of karyotypic evolution remain poorly understood.

In the course of evolution, snakehead fishes developed accessory air-breathing organs, which allows them to do without water for a long time and migrate over land to colonize new habitats (Sayer 2005; Bressman et al. 2019). These features have contributed to the distribution of snakeheads in a suitable climate zone, in particular species, such as *Channalucius* and *C.striata*, which have relatively large ranges in South Asia, including many freshwater habitats on both mainland and islands (Adamson et al. 2010; Tan et al. 2012). The wide geographical distribution associated with the lifestyle of *Channa* species, together with complex hydrographic and geological events in their habitats, led to the fragmentation and isolation of populations (Adamson et al. 2012; Tan et al. 2012; Robert et al. 2019), which in turn could have contributed to karyotypic changes. For example, the geographical separation of South Asian populations of *C.punctata* contributed to the fixation of various types of chromosomal rearrangements in different parts of the range, which led to karyotypic variability from 2n = 32 (NF = 58–64) to 2n = 34 (NF = 64) (Ruma et al. 2006; Kumar et al. 2013; Rakshit et al. 2015). Interpopulation chromosomal variability found among the cytogenetically studied snakehead species has led to the assumption that lifestyle, fragmentation, and isolation of populations contribute to an increase in karyotypic diversity (Cioffi et al. 2015). In this regard, it is of interest to study karyotypes in previously unexplored small and/or isolated populations of widespread *Channa* species.

This study presents chromosome complements of *C.lucius* and *C.striata* from Phu Quoc Island and comparative cytogenetic analysis (chromosome number and karyotype composition) of the genus *Channa*. The trends in the karyotypic evolution of snakeheads and chromosomal diversification due to the isolation of island populations are discussed.

## ﻿Material and methods

**Figure 1.**
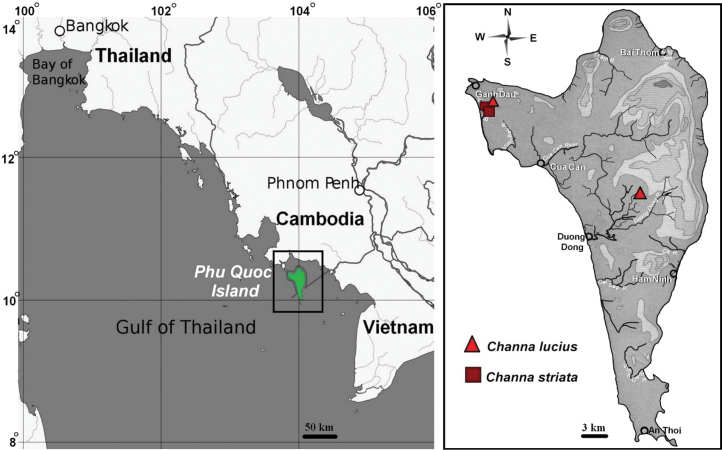
Map showing the location of Phu Quoc Island (left) and island details with *Channa* species collection sites (right).

Individuals of *Channa* species were collected from Phu Quoc Island (Gulf of Thailand, Vietnam) (Fig. 1) in December of 2011. Four individuals (two females and two males) of *C.lucius* (Cuvier, 1831) were karyotyped from the Bai Dai River and Duong Dong River basins (Fig. 1). Six individuals (two females and four males) of *C.striata* (Bloch, 1793) were karyotyped from the Bai Dai River basin (Fig. 1). Snakehead vouchers were deposited in the Southern Department of the Vietnam-Russian Tropical Center (Ho Chi Minh City). The total number of metaphase plates studied for each species was 65 and 82, respectively.

Chromosome preparations were obtained from the anterior part of the kidney according to previously published methods (Ojima and Kurishita 1980; Blanco et al. 2012) with the initial treatment of live fish with colchicine (injection of 0.08% solution into the spinal muscle). The anterior kidney tissue was incubated in 0.075 M KCl (hypotonic solution) for 24 min at 28 °C and fixed in 96% ethanol mixed with glacial acetic acid (3:1 ratio). Chromosome preparations made using standard air-drying techniques were stained with 5% Giemsa solution in phosphate buffer at pH 6.8 for 7 min. Mitotic chromosomes were analyzed under a microscope Leica DM 1000 with DFC 295 camera and LAS EZ software. Chromosomes were classified as metacentric (m), submetacentric (sm), subtelocentric (st), and acrocentric (a) according to their arm ratios (Levan et al. 1964). To determine the number of chromosome arms (NF), chromosomes of the m and sm groups were considered biarmed and those of the st/a group uniarmed. For statistical analysis of the results and data visualization, I used Excel 2021 software. The regression between the proportion of biarmed chromosomes and diploid chromosome number, and the Spearman correlation were calculated.

## ﻿Results and discussion

### ﻿Karyotypic diversity in *Channalucius* and *C.striata*

**Figure 2.**
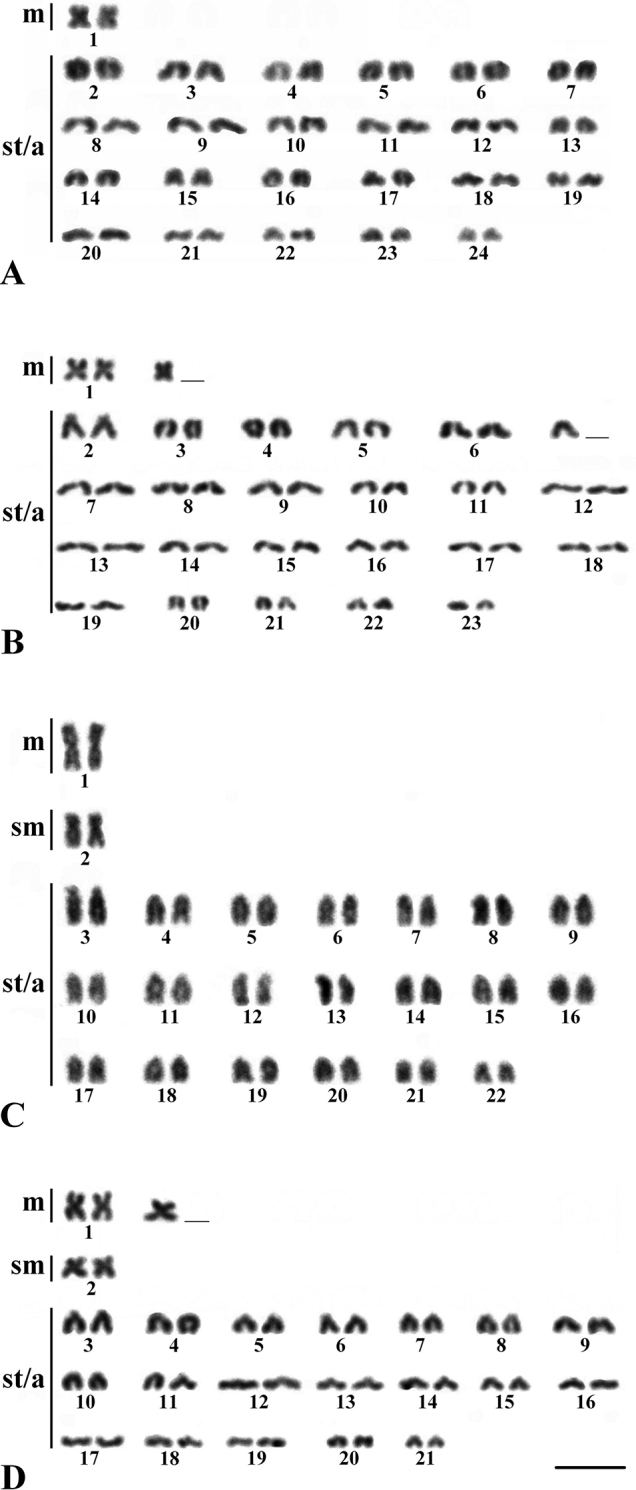
Karyotypes of *Channalucius* (**A, B**) and *C.striata* (**C, D**) from Phu Quoc Island. Numerals indicate the paired chromosomes. Scale bar: 5 μm.

For *C.lucius* from both studied localities of Phu Quoc Island, the same diploid number of 2n = 48 was characteristic, but a different karyotype composition. In individuals from the Bai Dai River basin, the karyotype consisted of 2 metacentric chromosomes (m) and 46 subtelocentric and acrocentric chromosomes (st/a), NF = 50 (Fig. 2A). The karyotype of individuals from the Duong Dong River basin consisted of 3m and 45 st/a, NF = 51 (Fig. 2B). *C.striata* from the Bai Dai River basin had karyotypes differing in the number of biarmed chromosomes with 2n = 44 (2m+2sm+40st/a) and 2n = 43 (3m+2sm+38st/a), NF = 48 (Fig. 2C, D). In the two studied species, no differences were observed between male and female karyotypes.

Comparative analysis of island and mainland populations of *C.lucius* showed interpopulation chromosomal variability. Populations from Thailand and Phu Quoc Island differed in the number of m-chromosomes (Table 1), which is probably due to pericentric inversion. Populations of *C.striata* were characterized by different levels of chromosomal polymorphism. Previous studies of mainland populations of *C.striata* have shown marked karyotypic variability ranging from 2n = 40 to 2n = 44 (Table 1). For a population from Northeastern Thailand, an atypical karyotype with 2n = 43 was found containing an unpaired large m-chromosome (Cioffi et al. 2015). It is assumed that individuals with 2n = 43 could have arisen both as a result of hybridization of two parental karyotypes with 2n = 44 and 2n = 42, and as a result of centric fusion of chromosomes in *C.striata* with 2n = 44 (Cioffi et al. 2015). In the polymorphic population of *C.striata* from Phu Quoc Island, the karyomorph with 2n = 43 was heterozygous for centric fusion. The maintenance and preservation of such a heterozygous state with an unpaired m-chromosome in different populations of *C.striata* may be evidence in favor of the fact that individuals with 2n = 43 produce viable gametes. Interestingly, on Phu Quoc Island, heterozygous karyotypes were also found in goby fish (Prazdnikov 2018). Previous studies have revealed the important role of heterozygous chromosomal rearrangements in maintaining karyotypic diversity in different groups of animals (Guerrero and Kirkpatrick 2014; Dobigny et al. 2017; Llaurens et al. 2017; Wellenreuther and Bernatchez 2018).

**Table 1.** Diploid chromosome number (2n), chromosome arm number (NF), karyotype structure, and collection site of *Channalucius* and *C.striata*.

Species	2n	NF	Karyotype structure	Locality	References
*C.lucius*	48	50	2m+46st/a	Northeastern Thailand (Bung Klua reservoir in the Roi-Et)	Khakhong et al. 2014
	48	52	2m+2sm+2st+42a	Thailand	Donsakul and Magtoon 1991
	48	52	4m/sm/st+44a	Southern Thailand (Tapi Basin)	Cioffi et al. 2015
	48	50	2m+46st/a	Vietnam (Phu Quoc Island, Bai Dai River Basin)	This study
	48	51	3m+45st/a	Vietnam (Phu Quoc Island, Duong Dong River Basin)	This study
*C.striata*	40	50	8m+2sm+2st+28a	India	Banerjee et al. 1988
	40	48	8m+6st+26a	India (Assam, Meghalaya)	Dhar and Chatterjee 1984
	40	48	8m+2st+30a	India (Imphal)	Rishi and Haobam 1990
	40	50	8m+2sm+30st/a	India (WB)	Manna and Prasad 1973
	40	58	8m+10sm+22a	India (Manipur)	Sobita and Bhagirath 2006
	40	48	6m+2sm+10st+22a	Northeastern India	Kumar et al. 2013; 2019
	42	48	6m+36st/a	Northeast Thailand (Khon Kaen, Mahasakam)	Supiwong et al. 2009
	44	46	2m+42a	China	Wu et al. 1994
	43	50	7m/sm/st+36a	Northeastern Thailand (Chi Basin)	Cioffi et al. 2015
	44	50	6m/sm/st+38a	Central and Southern Thailand (Chao Phraya Basin, Tapi Basin)	Cioffi et al. 2015
	44	48	2m+2sm+40a	Thailand	Donsakul and Magtoon 1991
	44	48	2m+2sm+40st/a	Vietnam (Phu Quoc Island, Bai Dai River Basin)	This study
	43	48	3m+2sm+38st/a		This study

The probable maximum age of isolation of Phu Quoc Island from the Cambodian mainland is about ten thousand years when sea levels rose after the end of the last glacial period (Kuznetsov and Kuznetsova 2011). The short-term isolation of the island populations of *C.lucius* and *C.striata* probably contributed either to the appearance of chromosomal polymorphism or its maintenance due to at least two types of chromosomal rearrangements. Further cytogenetic studies of these two snakehead species from different river basins of the island and an increase in the sample size will most likely reveal an even greater range of variability in the number of 2n and NF.

### ﻿Karyotypic evolution in *Channa* species

**Figure 3.**
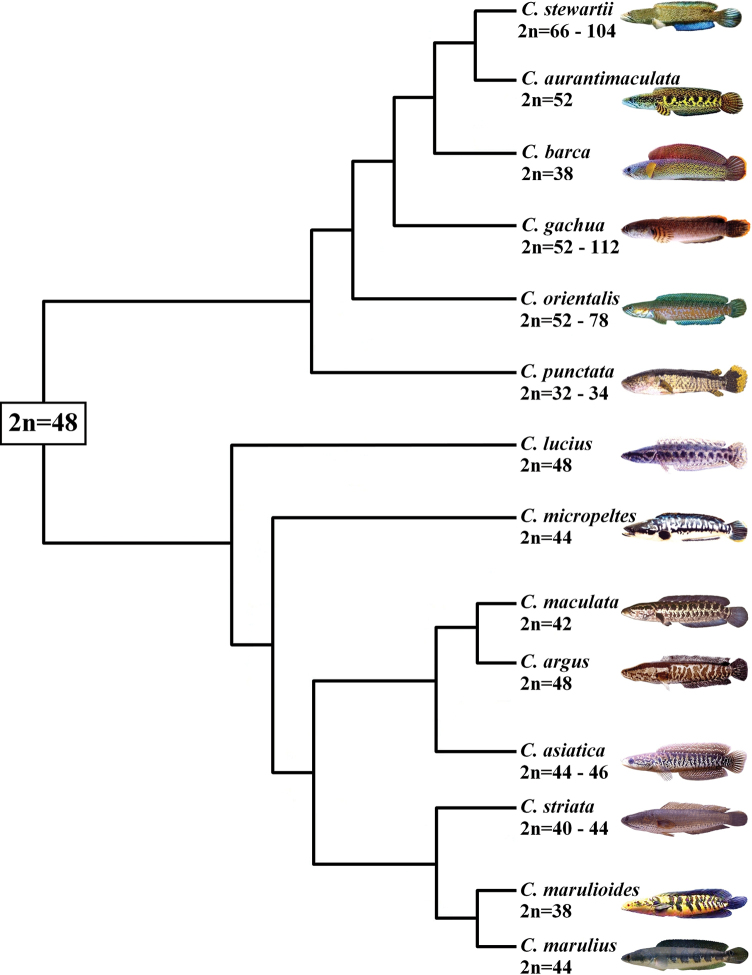
Phylogenetic tree of the cytogenetically studied *Channa* species (based on Kumar et al. 2019 with modifications and additions) indicating the putative ancestral karyotype (in a rectangle) and range of variability of diploid chromosome numbers.

**Figure 4.**
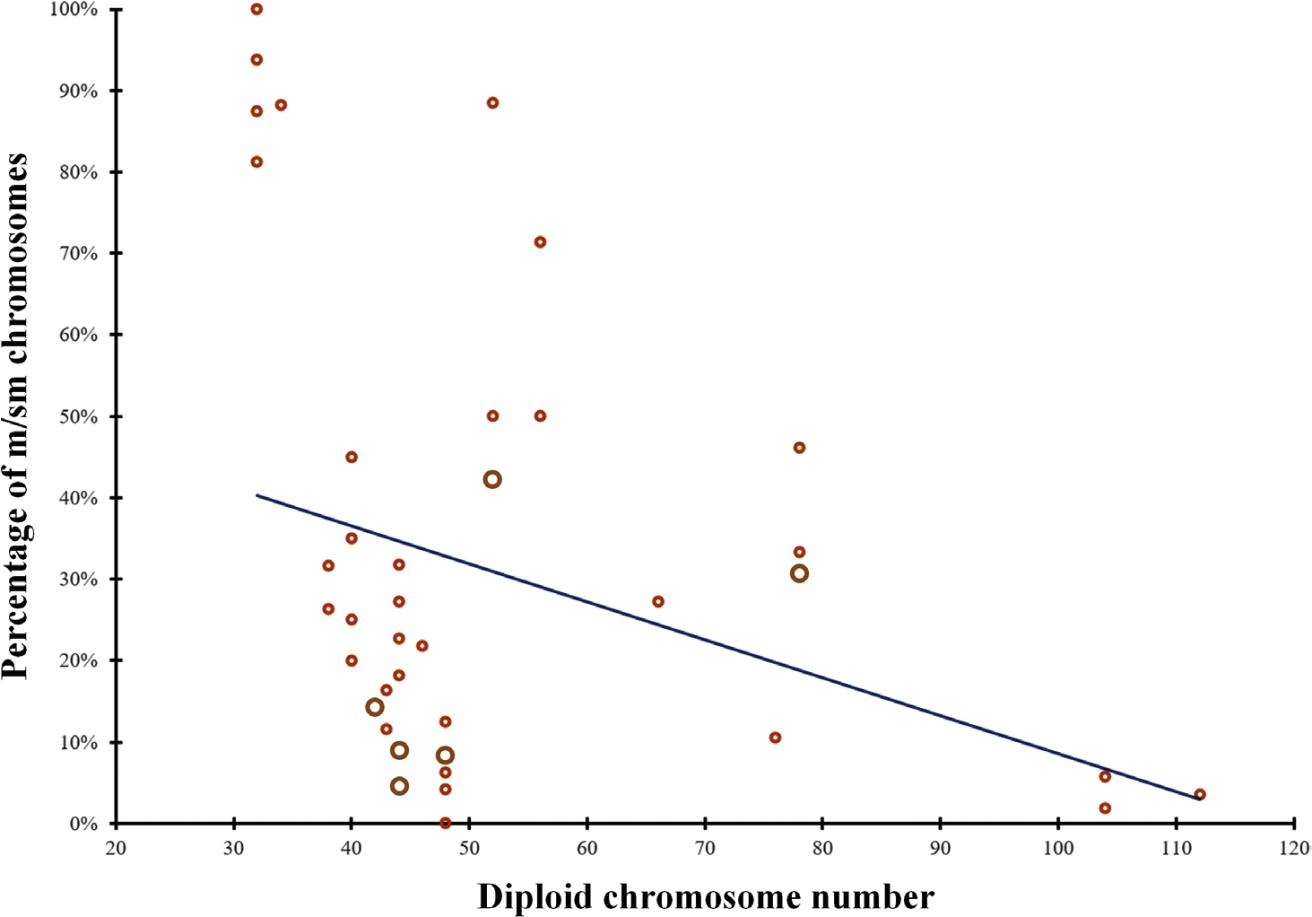
Scatter plot of a diploid chromosome number and proportion of metacentric/submetacentric chromosomes (m/sm) with overall regression line for the genus *Channa*. The diameter and color of a circle indicate the number of species from 1 to 2.

An analysis of cytogenetic data (Dhar and Chatterjee 1984; Banerjee et al. 1988; Rishi and Haobam 1990; Sobita and Bhagirath 2006; Tanomtong et al. 2014; Cioffi et al. 2015; Kumar et al. 2019) indicated that the karyotypic evolution in *Channa* species occurred in different directions and at different rates, which led to a wide chromosomal diversity from 2n = 32 to 2n = 112 (Fig. 3). The proportion of biarmed chromosomes in the karyotype varies widely from 0% to 100%. The regression between the proportion of biarmed chromosomes in the karyotype and the diploid number is y = -0.0046x + 0.551 (R² = 0.102), and the Spearman correlation is R_s_ = -0.28 (Fig. 4). The weak correlation between the two variables (2n and proportion of m/sm chromosomes) is apparently due to chromosomal rearrangements that affected the trends of karyotypic evolution in the genus *Channa*.

**Figure 5.**
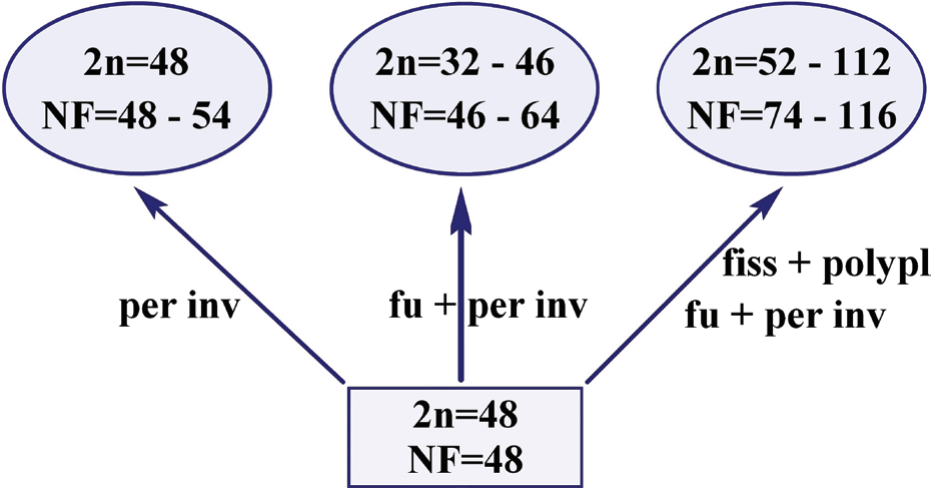
The trends of karyotypic evolution for the genus *Channa*. The thick blue arrow shows the most likely main trend in karyotypic evolution. The lower rectangle shows the ancestral karyotype. fu – centric fusions, fiss – centric fissions, per inv – pericentric inversions, polypl – polyploidization.

The probable ancestral karyotype of snakeheads consisted of 48 uniarmed chromosomes, which would require a minimum number of chromosome rearrangements during the karyotype transformation of the number of *Channa* species. Among the cytogenetically studied species, *C.argus* and *C.lucius* have a karyotype with 2n = 48; the latter is also characterized by plesiomorphic features, such as the gular scales, which is absent in most species of Asian snakeheads (Li et al. 2006). The main trend of karyotypic evolution of *Channa* species is associated with a decrease in the number of chromosomes due to centric fusions (Robertsonian translocations) and an increase in the number of chromosome arms due to pericentric inversions (Fig. 5). As a result, in some populations of *C.punctata*, symmetrical karyotypes with 2n = 32 appeared, consisting exclusively of biarmed chromosomes (Dhar and Chatterjee 1984; Rakshit et al. 2015). Another direction of karyotype transformation is associated with an increase in the number of chromosomes as a result of centric fission and polyploidization, followed by an increase in the proportion of m/sm chromosomes as a result of centric fusions and pericentric inversions (Fig. 5). The huge variability in 2n of *C.gachua* (2n = 52–112), *C.orientalis* (2n = 52–78), and *C.stewartii* (2n = 66–104) indicates both the possibility of ploidy change in different populations (Cioffi et al. 2015; Kumar et al. 2019) and their co-evolution (Tanomtong et al. 2014). An additional direction of karyotype transformation in *Channa* is associated with an increase in the proportion of biarmed chromosomes without changing 2n (mainly due to pericentric inversions) (Fig. 5). It is likely that another mechanism, such as centromere repositioning, could also be involved in alterations in chromosome morphology (Sobita and Bhagirath 2006; Rakshit et al. 2015).

Chromosomal rearrangements, which involve karyotypic structural changes such as inversions and fusions, may play an important role in the adaptive evolution of fish (Wellband et al. 2019; Cayuela et al. 2020). Rearrangements disturb homologous chromosome pairing during meiosis, resulting in tight linkage among genes encoding adaptations (for example, to salinity gradient and temperature) within rearranged regions (Barth et al. 2017; Wellenreuther and Bernatchez 2018). Such chromosomal rearrangements suppress recombination, and important functional genes are inherited together, which may contribute to adaptive population divergence (Barth et al. 2017). *C.gachua* is known to be well adapted to survive in a variety of habitats, in higher mountain areas with fluctuating climates, and has more resistance than other *Channa* species (Courtenay and Williams 2004; Tanomtong et al. 2014), which may be due to the high level of karyotypic variability.

The proposed expansion of the ranges of modern taxa of Asian snakeheads at the Miocene/Pliocene boundary, combined with climatic fluctuations, led to repeated isolations of populations, especially continental islands, and secondary contacts between them (Adamson et al. 2010; Tan et al. 2012; Wang et al. 2021), which probably influenced the chromosome diversification in the genus *Channa* with the formation of intrapopulation and interpopulation chromosomal variability. The karyotypic diversity of snakeheads can also increase as a result of hybridization, which is possible even between species that differ in the number of 2n and NF (Ou et al. 2018). Obviously, further studies will make it possible to reveal even greater karyotypic diversity associated with the appearance of biarmed chromosomes within the framework of the main trend in the karyotypic evolution of *Channa* species.
